# Advantageous Extraction, Cleanup, and UHPLC-MS/MS Detection of Patulin Mycotoxin in Dietary Supplements and Herbal Blends Containing Hawberry from *Crataegus* spp.

**DOI:** 10.1155/2019/2159097

**Published:** 2019-02-06

**Authors:** Anna Przybylska, Grzegorz Bazylak, Robert Kosicki, Iwona Altyn, Magdalena Twaruzek, Jan Grajewski, Anna Soltys-Lelek

**Affiliations:** ^1^Department of Pharmaco-Bromatology and Molecular Nutrition, Faculty of Pharmacy, Collegium Medicum, Nicolaus Copernicus University, Jagiellonska 13, PL-85067 Bydgoszcz, Poland; ^2^Department of Physiology and Toxicology, Institute of Experimental Biology, Faculty of Natural Sciences, Kazimierz Wielki University, Chodkiewicza 30, PL-85064 Bydgoszcz, Poland; ^3^Ojcow National Park, Ojcow 9, PL-32045 Suloszowa, Poland

## Abstract

Patulin (PAT) is a highly genotoxic mycotoxin still found as the common contaminant of various kinds of spoiled fruits and related commodities which are often endorsed as the health-enhancing products. Thus, a fast and convenient liquid-solid extraction followed by a solid-phase cleanup with the MycoSep®228 AflaPat multifunctional column was used for the highly efficient isolation of PAT with an average recovery of 112.7% from commercial dietary supplements and herbal blends formulated with dried hawberry. Analysis of the PAT content was carried out using gradient elution with a Synergi Polar C18 column (150 × 2 mm, 4 *μ*m) and UHPLC system equipped with a mass spectrometer. PAT was detected in all (*n*=14) commercial single-component dietary supplements formulated with dried hawberry belonging to *Crataegus monogyna* and/or *Crataegus laevigata*. Similarly, PAT was detected in 67% of the studied multicomponent commercial herbal blends (*n*=6) that contained—in addition to hawberry—different amounts of apple, chokeberry, elderberry, hibiscus, or mallow. Moreover, the PAT content was determined in the hawberry collected from the mature wild hawthorn trees belonging to three botanical species, *Crataegus monogyna* Jacq., *Crataegus laevigata* (Poiret) DC, and *Crataegus rhipidophylla* Gand, growing in the recreational forest areas and in the law-protected state national forest park in Poland. In conclusion, to prevent PAT accumulation and reduce the health risk of consumers in globalizing markets, the implementation of improved cultivation/processing practices of hawthorn trees and hawberry as well as increased analytical control related to the presence of PAT in dietary supplements and herbal blends formulated with fresh, dried, or frozen hawberry should be urgently recommended.

## 1. Introduction

The use of hawthorn fruit (also called hawberry) and hawthorn inflorescences in folk and official medicine has a long tradition in many European countries, and currently, hawberry-derived dietary supplements, herbal medicines, and pharmaceuticals are commonly used by people with cardiovascular problems [1]. According to the recommendation of the European Pharmacopaeia [2], dietary supplements and herbal blends can be mainly formulated with use of dried hawberry, commonly described as the “false fruit” of hawthorn trees belonging to *Crataegus monogyna* Jacq. (Lindm.) or *Crataegus laevigata* (Poir.) DC (syn. *C. oxyacantha* L.), as well as a mixture of hawberry collected from both mentioned *Crataegus* spp. species or, alternatively, hawberry from the hybrids of these species. In addition, the European Pharmacopaeia [2] recommends a minimum concentration of 0.06% of procyanidines, expressed as cyanidin chloride (C_15_H_11_ClO_6_), in such hawthorn-based therapeutic products.

Traditionally, hawberry is harvested by many citizens and village inhabitants for their own needs and used in folk medicine for treatment of hypertension, obesity, and menopause and improving memory in Mexico [3], Poland [4], and Portugal [5]. Numerous studies have confirmed that standardized *Crataegus* spp. extract WS® 1442 obtained from carefully planted, harvested, and stored hawberry is healthy and safe and has highly beneficial effects in various groups of patients [6]. The review by Zorniak et al. [7] suggested that standardized extract WS® 1442 can be successfully used as an addition to optimal treatment of chronic heart failure in clinical conditions. Research conducted by Veveris et al. [8] confirmed that the oral treatment of male Wistar rats with the standardized *Crataegus* spp. extract WS®1442 protects against the cardiovascular side effects following arrhythmias and heart reperfusion and can prevent myocardial dysfunction.

The numerous beneficial outcomes of hawberry and their hot water extracts and infusions are due to the presence of active compounds such as flavonoids, especially the oligomeric proanthocyanidins [9]. The composition of procyanidins in hawberry from each of the *Crataegus* spp. is very characteristic, and it has been suggested that these compounds are more bioactive with an increased degree of polymerization [8].

In many European countries, the most common species of hawthorn trees are *Crataegus monogyna* Jacq., *Crataegus laevigata* (Poiret) DC, *Crataegus* × *macrocarpa* Hegetschw, and *Crataegus rhipidophylla* Gand. [10, 11], while in Asia, the species of *Crataegus azarolus* L., *Crataegus pentagyna* Willd., and *Crataegus pinnatifida* Bunge have been most frequently harvested [12]. The hawberries from each of the species of the *Crataegus* genus differ in their content of active compounds, e.g., flavonol-*O*-glycoside and vitexin-2″-*O*-rhamnoside. Edwards et al. [13] found that the content of flavonol-*O*-glycoside was in the range of 0.29–0.76 mg/g in the fruit of species *Crataegus cuneata* compared to 1.29–3.45 mg/g in the fruit of species *Crataegus monogyna*. Similarly, the content of vitexin-2″-*O*-rhamnoside was in the range of 0.021–0.023 mg/g in the hawberry of *Crataegus pinnatifida*, and this compound was not detected in the hawberry from the species *Crataegus aronia* var. *aronia*. However, in *Crataegus monogyna*, the content of these flavonoids was up to 0.148 mg/kg [13].

Patulin (4-hydroxy-4,6-dihydrofuro[3,2-c]pyran-2-one) is a secondary polyketide metabolite produced as a mycotoxin by several species of mold such as *Penicillium*, *Aspergillus,* and *Byssochlamys* [14–17]. It has been postulated that the fungal biosynthesis of PAT takes place in an enzymatic cascade involving 10 steps in which individual enzymes could be activated consecutively as the newly synthesized product is being metabolized [18, 19]. Regardless of the exact fungal mechanism of PAT biosynthesis, studies on the cytotoxicity of PAT on a variety of animal organisms showed it causes mitochondrial dysfunction, activates apoptotic signaling pathways, and induces reactive oxygen species-mediated oxidative cell damage [17]. Many studies have described immunotoxic, neurotoxic, dermotoxic, or teratogenic effects of PAT on animals and humans [19, 20]. However, recently, according to the International Agency for Research on Cancer (IARC), PAT was classified as a mycotoxin without carcinogenic properties [21].

The major sources of PAT in the common human diet are apples, apple juice, pears, grapes, and strawberries [22]. The World Health Organization (WHO) recommends limiting the maximal PAT content in apple to 50 *µ*g/kg, in apple juice to 25 *µ*g/kg, and in baby food to 10 *µ*g/kg. The Joint Expert Committee on Food Additives of the World Health Organization (JECFA) recommended that daily human exposure to PAT should be reduced to 0.4 *µ*g/kg body weight per day [20, 23]. In Europe, in addition to being found in apples, PAT has been found in tomatoes, pears, quinces, apricots, cherries, black currants, and blue cheeses [20, 24]. In previous years, the number of studies describing the occurrence of PAT in products containing hawberry is still limited. However, some recent data on PAT determination in hawberry-derived beverages [25], hawberry juice [26], hawberry slices, including dried, baked, and charred [27], and dried hawberry products in circular flakes, rolls, strips, and dessert forms [28], have been reported from China. To our best knowledge, for Europe, there are still no papers describing the content of PAT in freshly harvested or stored hawberry or in dietary and medicinal products containing dried or frozen hawberry.

The aim of this study was to evaluate the PAT content in a series of typical, available-in-Poland, commercial dietary supplements and herbal blends containing dried hawberry that have been used by subjects suffering from cardiovascular diseases and hypertension. In addition, the content of PAT in representative samples of wild freshly harvested and naturally dried or frozen hawberry have been determined.

## 2. Materials and Methods

### 2.1. Samples

Twenty commercially available therapeutic products and 21 harvested samples (batches) containing hawberry were analyzed in this study. Commercial products in the form of dietary supplements (*n*=14) and herbal blends (*n*=6) were purchased from supermarkets and pharmacies in Bydgoszcz (Poland) in September 2016. These medicinal products contained hawberry originating from organic farms (*n*=3) and commercial plantations (*n*=17) located in various regions of Poland, as shown in Table 1. The dietary supplements contained exclusively dried hawberry (group I), while herbal blends (group IA) contained a mixture of dried hawberry and different dried parts of various medicinal plants, i.e., hawthorn inflorescence, hibiscus flower, apple fruit, chokeberry, elderberry, rosehip, lavender flower, horsetail herb, or mallow flower. The weight content of the dried hawberry in these kinds of commercial herbal blends was in the range of 40–60%. Dietary supplements and herbal blends studied here are commonly consumed in Poland in the form of home-prepared water infusions where portion of 2.5 g of these hawberry-derived products with 200 mL of boiling water (100°C) in time of 10 min is used [15, 20].

Manufacturers provided information about the genus species of hawthorn trees from which the hawberry was used to produce their commercial dietary supplements and herbal blends (Table 1). The six different samples (batches) of hawberry *Crataegus laevigata* and *Crataegus rhipidophylla* (group II in Table 1) were collected, air-dried naturally, and stored at +20°C from the mature wild hawthorn trees located in the Ojcow National Park located in south part of Poland. Fifteen batches of hawberry (group III in Table 1) were harvested from the mature wild hawthorn trees identified as the *Crataegus monogyna* species and located in two different places in the Fordon and Bartodzieje districts of Bydgoszcz (north part of Poland). All of these hawberry batches from Bydgoszcz were stored at −20°C prior to the PAT content analysis performed in December 2016. Healthy and undamaged hawberry were harvested in Ojcow and Bydgoszcz in September/October 2016. The genus and species of the mature wild hawthorn trees and their fruits were identified using a set of specific morphological data with the kind expertise support of Dr. Anna Sołtys-Lelek from Ojców National Park, Poland.

### 2.2. Chemicals and Reagents

Internal standards of the nonlabeled patulin (PAT) and the isotopically labeled ^13^C patulin (^13^C-PAT) were obtained from, respectively, Sigma-Aldrich (St. Louis, MO, USA) and Biopure (Guntramsdorf, Austria). The crude methanolic solutions (0.5 mg/mL) of each of these standards were stored at 4°C. Ultrapure acetonitrile, methanol, and ammonium acetate were obtained from Merck (Darmstadt, Germany). Deionized water (80 MΩ) used in the analysis was prepared using a Simplicity 185 water purification system (Millipore, Bedford, MA, USA).

### 2.3. Extraction Procedure of PAT

Four grams of each studied product with hawberry were carefully milled and homogenized to a fine powder with the final fineness of approximately 10 *μ*m using a mortar grinder. Next, this powdered sample was dissolved in 16.0 mL of acetonitrile : water (80 : 20, v/v) mixture, vigorously shaken for 45 min, and centrifuged at 3500 × *g* for 10 min. Then, 8.0 mL of this obtained sample extract (supernatant) was passed over 30 sec by the push-through-type solid-phase cleanup/filtration column MycoSep®228 AflaPat (Romer, Newark, DE, USA). The 4.0 mL of purified eluate was transferred into a conical vial and evaporated to dryness under a gentle stream of nitrogen at 45.0°C. The residue was redissolved in 1.0 mL of a methanol : water (1 : 4, v/v) mixture by sonication and then filtered by a 0.22 *µ*m PTFE syringe filter (Macherey-Nagel, Düren, Germany). To the total volume of 180.0 *µ*L of this purified extract was added 20.0 *µ*L of isotopic ^13^C-labeled PAT (^13^C-PAT) (or, alternatively, nonlabeled PAT) as the internal standard solution at a concentration of 0.5 mg/mL. Then, this final sample solution was placed in a conical vial for the automatic sampler to perform the analysis.

### 2.4. UHPLC-MS/MS Analysis of PAT

A Shimadzu Nexera LC system (Shimadzu, Kyoto, Japan) equipped with a 5500 Q-TRAP triple quadrupole mass spectrometer (Sciex, Framingham, MA, USA) was used in the analysis. Separation of PAT was done with the use of a Synergi Polar C18 column (150 × 2 mm, 4 *µ*m) supplied by Phenomenex (Torrance, CA, USA). The binary mobile phase was a combination of solvent A (10 mM ammonium acetate in water) and B (10 mM ammonium acetate in methanol), applying the following gradient program: 0.0–1.0 min, linear from 0.0 to 10.0% B; 1.0–7.0 min, linear from 10.0 to 90.0% B; 7.0–8.0 min, 90.0% B; 8.1–12.0 min, 10.0% B. The flow rate of the mobile phase was 1.0 mL/min, and the injection volume of the analyzed sample was 5.0 *µ*L. Curtain- and collision-induced dissociation gas flow was set at 80 and 30 psi, respectively. The ion source temperature was 600°C. The ion spray voltage was set to 4500 V. Declustering and cell exit potential were set to −85 V and −7/−9 V for nonlabeled PAT and −95 V and −13/−13 V for ^13^C-labeled PAT. Collision energy was set at −12 and −16 V for, respectively, PAT and ^13^C-PAT. The multiple reaction monitoring (MRM) was operated with dwell time 200 ms at *m*/*z* 153/109.1 and 153/81 for PAT and *m*/*z* 160/115 and 160/86.1 for ^13^C-PAT in the electrospray ionization (ESI) negative ion mode.

### 2.5. Fragmentation Pathways of PAT and ^13^C-PAT

**Figure 1:**
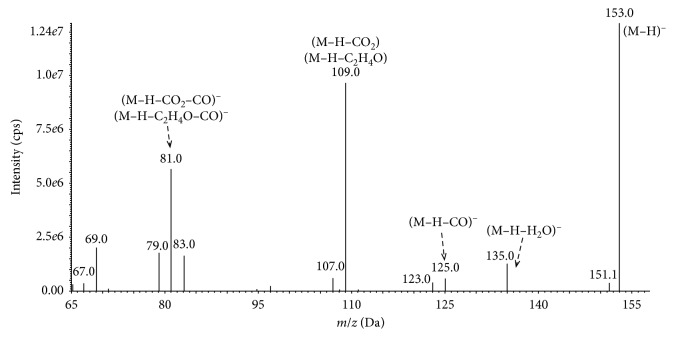
Mass spectrum showing the deprotonated molecular ion [M-H]^−^ of PAT at *m*/*z* 153.0.

The probable fragmentation path of the nonlabeled PAT was determined from the ESI-MS/MS mass spectrum, as presented in Figure 1, by identifying differences of the *m*/*z* values. The mass spectra consists of deprotonated molecular ion [M-H]^−^ of PAT at *m*/*z* 153.0 and gives much structural information. The outstanding intensity of the pseudomolecular ion [M-H]^−^ of PAT may indicate the high stability of this compound.

**Figure 2:**
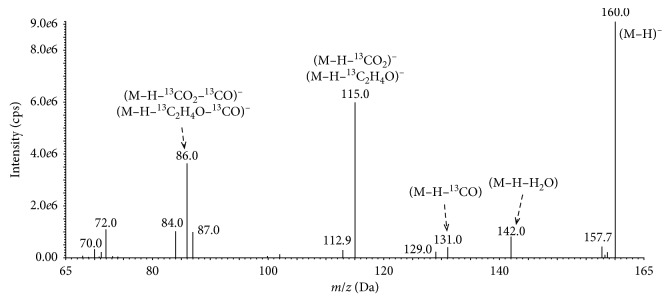
Mass spectrum showing the deprotonated molecular ion [M-H]^−^ of ^13^C-labeled PAT at *m*/*z* 160.0.

Among the eliminated groups, we could identify water (−18 Da), carbon monoxide (−28 Da), carbon dioxide (−44 Da), acetaldehyde (−44 Da), and fragments of ions with a mass of 72 Da corresponding to compounds with a molecular formula C_2_O_3_ or C_3_H_4_O_2_. The ion at *m*/*z* 109 indicates loss of carbon dioxide (CO_2_) or acetaldehyde (C_2_H_4_O) by the parent ion [M-H]^−^ of PAT. Due to being the same molecular weight, it is difficult to separate these two compounds. These results are in good agreement with previously published research on the PAT mass spectra and earlier studies on the fragmentation of PAT [17, 29, 30]. The observed path of fragmentation of the ^13^C-labeled PAT, as presented in Figure 2, was very similar to the fragmentation of the nonlabeled PAT.

### 2.6. Method Validation

The analytical procedure developed here was validated according to the ICH guidelines for its specificity, linearity, accuracy, precision, limit of detection (LOD), limit of quantification (LOQ), and robustness [31]. Quantification of UHPLC results was performed by comparing peak areas for each analyzed sample to calibration curves of PAT standards [32]. Recovery and precision of the method was evaluated by analyzing fortified samples at different PAT or ^13^C-PAT concentrations in triplicate. Correlation coefficients (*R*) were calculated to estimate linearity of peak areas relative to the PAT standards. Internal standards (^13^C-PAT or PAT) were used to correct for analytical recovery and for matrix effects in time of MS/MS detection. In this SPE-UHPLC-MS/MS procedure, the extraction recovery for the spiked PAT at concentrations of 50.0 and 100.0 *µ*g/kg was 112.7 and 109.4%, respectively. The concentration linearity range for PAT was 2.0 to 100.0 *µ*g/kg (*R* = 0.9987). Limits of detection (LOD, signal-to-noise ratio of 3) and quantification (LOQ = 3 × LOD) for PAT were, respectively, 1.0 and 3.0 *µ*g/kg. LOD and LOQ were calculated by spiking samples at low concentrations with PAT (1, 5, 10, 20, 50, and 100 *µ*g/kg) and subjecting them to all steps of sample preparation. The intraday (*n*=6) and interday (*n*=15) precision (% RSD) of this method of PAT determination were, respectively, in the range of 5.2–10.6% and 7.8–12.4%.

### 2.7. Statistical Analysis

All analyses were carried out in triplicate. The obtained results were evaluated with Statistica v.9 (StatSoft, Tulsa, OK, USA). The results are presented as the mean ± standard deviation (Table 2). In all of these analyses, we examined the outcome of the mean content of PAT using Student's *t*-test and *p* value ≤ 0.05 was considered as significant. In hierarchical clustering calculation, the Ward agglomeration procedure as a grouping method and Euclidean distance as a function of the distance was applied, and results are displayed graphically using a dendrogram (classification tree). Differences in profile among subclusters were verified with the nonparametric Mann–Whitney *U* test at *p* ≤ 0.05 as significant.

## 3. Results and Discussion

### 3.1. UHPLC-MS/MS Analysis

**Figure 3:**
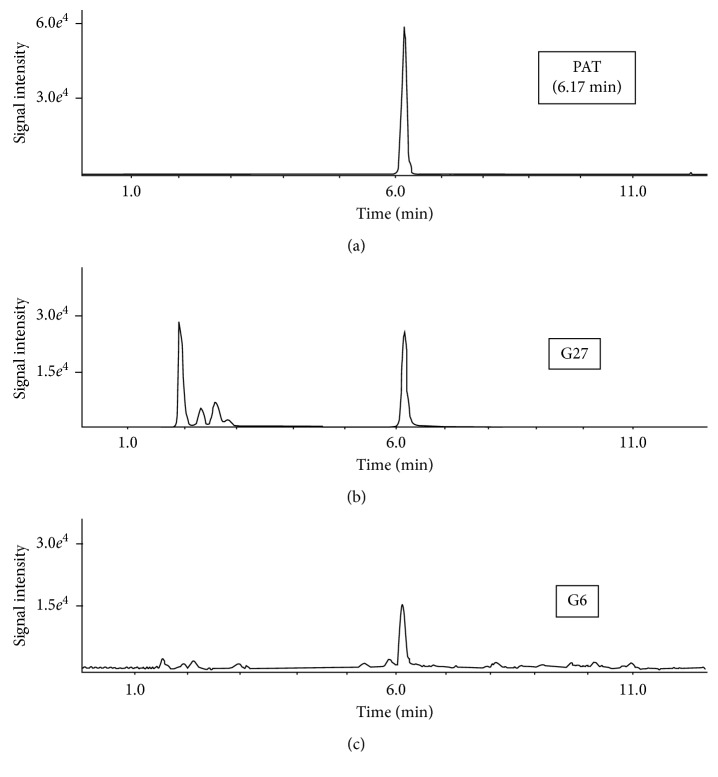
(a) Chromatograms of the standard solution of PAT referring to concentration of 30 *µ*g/kg; (b) dietary supplement G27 with an average PAT content of 15.6 *µ*g/kg; (c) dietary supplement G6 with an average PAT content of 9.1 *µ*g/kg.

The representative chromatograms obtained during UHPLC-MS/MS analyses of PAT content in the dietary supplements, herbal blends, and fresh samples are shown in Figure 3. The identity of PAT was confirmed by comparing the electrospray ionization (ESI) MS/MS spectrum of the peak eluting at the retention time observed earlier for the nonlabeled and isotope-labeled PAT standards (Figures 1 and 2). Reference negative ion mode ESI MS/MS spectra of PAT standards were acquired and interpreted as described Section 2.5. In addition, PAT was confirmed in the contaminated hawberry products after spiking the extract with an equivalent amount of PAT and checking the symmetry of the parent eluting peak at the exact retention time of this compound.

Compared to the work of Li et al. [25] on determination of PAT in the hawberry products with the Xterra C18 column and isocratic elution using tetrahydrofuran-water (0.8 : 92.2, v/v) as the mobile phase, the retention time of PAT in the gradient UHPLC conditions was practically the same (i.e., 5.80 vs. 6.17 min); however, the LOD for the PAT achieved in the former method, operating with UV detection at 276 nm, was only 8.0 *µ*g/kg compared to 1.0 *µ*g/kg offered by our method. Similarly, compared to the retention time of PAT observed in the isocratic HPLC method used to analyze hawberry products reported by Zhou et al. [26] with a Venusil C18 column and acetonitrile-water (5 : 95, v/v) as the mobile phase, we found in our gradient HPLC conditions a nearly sevenfold shorter retention time for PAT, and moreover, the LOD for PAT reported in the former method with diode array detection was 3.99 *µ*g/kg compared to 1.0 *µ*g/kg found using our procedure. The separation efficiency of the gradient UHPLC method proposed in our study is also superior to the isocratic HPLC procedure reported by Ji et al. [28] for the study of hawberry products using an Xbridge C18 column and acetonitrile-water (10 : 90, v/v) mobile phase, in which the retention time of PAT was 8.18 min and the LOD for PAT with UV detection at 276 nm was in the range 2.6 to 7.5 *µ*g/kg. In addition, the retention time of PAT in our gradient UHPLC procedure was nearly twofold higher than that obtained using the gradient HPLC method reported by Xiang et al. [27] for determination of PAT in dried hawberry-derived medicinal products, where a Shiseido Capcell PAK C18 MGIII column was used with 5.0 mmol/L ammonium acetate in methanol as the mobile phase. In the same report by Xiang et al. [27], the use of the ESI MS/MS detection mode offered an LOD of PAT of 3.0 *µ*g/kg.

Replacement of the acetonitrile used in the cleanup/filtration step with a MycoSep®228 AflaPat multifunctional column by Li et al. [25] and Zhou et al. [26] and with a ProboFast®228 multifunctional column applied by Ji et al. [28] with acetonitrile-water (80 : 20, v/v) in our procedure resulted in an improved average recovery of PAT up to 112.7% at a spiked level of 50.0 *µ*g/kg, and the extraction method accuracy and precision of PAT determination in hawberry products was quite comparable to these three mentioned earlier reports. The satisfactory average extraction recovery of PAT observed in our study (112.7%) was highly improved compared to the rather low average recovery of PAT (i.e., 64.0%) from hawberry products as reported by Xiang et al. [27] after use of the ultrasonically supported water-ethyl acetate liquid-liquid extraction.

**Figure 4:**
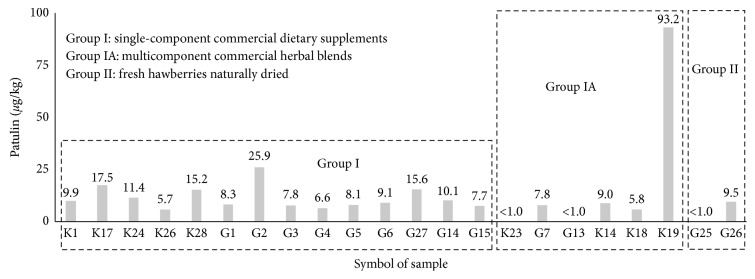
Comparison of average content of PAT in the hawberry-derived commercial products and freshly harvested samples. The all frozen hawberry harvested in Bydgoszcz, Poland (group III), and the four hawberry samples collected in Ojcow, Poland (group II), where the PAT content was below 1.0 *μ*g/kg have been omitted for clarity (Tables 1 and 2).

The content of PAT in the studied dietary supplements, herbal blends, and wild hawberry is shown in Table 2 and Figure 4. PAT was found in 47% of the 41 studied products and samples, which means that PAT was identified in 19 analyzed items, with an average PAT concentration of 14.9 ± 19.5 *µ*g/kg (range 5.7/93.2 *µ*g/kg, median concentration of PAT was 9.10 *µ*g/kg). The highest average content of PAT was found in the herbal blend numbered K19 (Table 1), where it reached the value of 93.2 *µ*g/kg, while the lowest concentration of PAT was below the limit of detection (LOD = 1.0 *µ*g/kg) in all of the samples of frozen wild hawberry from Bydgoszcz (samples F1–F15 in Table 1 and group III in Table 2), the five naturally dried wild hawberry from Ojcow (G23, G24, G25, G27, and G28 in Table 1), and the two herbal blends K23 and G13 (Table 1 and Figure 4).

The results showed that 100% of the commercial dietary supplements (*n*=14, group I in Table 1) formulated with hawberry from *C. monogyna*/*C. laevigata* trees were contaminated by PAT with an average concentration of 11.4 ± 5.5 *µ*g/kg and a range of 5.7 to 25.9 *µ*g/kg. However, in the group of studied herbal blends (*n*=6, group IA, Table 1), only 67% of products were contaminated by PAT with an average concentration of 15.7 ± 31.6 *µ*g/kg and a range of <LOD to 93.2 *µ*g/kg. In the one sample of wild hawberry harvested in Ojcow (G26), the average concentration of PAT was 9.5 *µ*g/kg, as shown in Figure 4, which imply that only 17% of these samples of hawberry have been contaminated with PAT at average content of 2.4 ± 3.4 *µ*g/kg (compare Table 2).

For comparison, in the medicinal dried hawberry cultivated with unknown *Crataegus* spp. species that were purchased from the local pharmacies in Jinan city (China), the average content of PAT was 15.1 *µ*g/kg, with a range from 4.6 to 38.8 *µ*g/kg, as determined by Xiang et al. [27]. A slightly lower content of PAT was found in 10% of commercial, dried hawberry products purchased from various supermarkets in Hangzhou (China). The range of concentration of PAT in these products was 5.1/11.1 *µ*g/kg [28]. In the hawberry beverages, PAT was found in 14% of the analyzed samples, and the average content of PAT was 116.3 *µ*g/kg, with a range of 19.8 to 206.9 *µ*g/kg [25]. The lower content of PAT at 12.3 *µ*g/kg was determined in the one sample of hawberry juice purchased in a supermarket in Beijing (China) [26].

Our results showed that only in the one studied product, i.e., herbal blend K19, was the average concentration of PAT nearly twofold higher than the WHO acceptable limit of PAT for apples, i.e., 93.2 versus 50.0 *µ*g/kg. Next, in the dietary supplement G2 and the herbal blend K19, the content of PAT exceeded the WHO acceptable upper limit of 25 *µ*g/kg that is recommended for clear and cloudy apple juices. Our results also showed that, in the set of seven analyzed dietary supplements and herbal blends (K17, K24, K28, G2, G27, G14, and K19), the content of PAT was significantly higher than 10 *µ*g/kg, which is the acceptable upper limit determined by WHO for baby food, which could also be used as the reference upper limit for hawberry-derived therapeutic products consumed by older humans and convalescent subjects.

Considering the place of origin, i.e., the province of Poland specified in Table 1, we observed that the average content of PAT in the PAT-contaminated samples (*n*=19) was significantly different and decreased in this order: Lodzkie Province (23.5 ± 34.2 *µ*g/kg, *n*=6), Podlaskie Province (13.9 ± 4.3 *µ*g/kg, *n*=3), Wielkopolskie Province (11.9 ± 9.4 *µ*g/kg, *n*=4), Swietokrzyskie Province (11.4 ± 1.4 *µ*g/kg, *n*=1), Malopolskie Province (5.2 ± 4.1 *µ*g/kg, *n*=3), and Lubulskie Province (4.9 ± 5.7 *µ*g/kg, *n*=2). These data suggest that hawberry harvested and processed by manufacturers located in central and northeast parts of Poland (Lodzkie Province and Podlaskie Province, respectively) had nearly 3- to 4-fold higher concentrations of PAT than the hawberry grown and processed in the south and southwest parts of Poland (Malopolskie Province and Lubuskie Province). In dietary supplement G15 supplied from an Internet store, the average content of PAT was 7.7 *µ*g/kg (Figure 4 and Table 2).

Our results also suggested that noncontrolled addition of apple (*Malus domestica* Borkh.), chokeberry, (*Aronia melanocarpa* (Michx.) Elliott), and elderberry (*Sambucus nigra* L.) accompanied by flowers of ethnoveterinary plant hibiscus (*Hibiscus sabdariffa* L.) or garden plant mallow (*Althaea rosea* L.) by manufacturers to the hawberry-derived herbal blends G7, K19, K14, and K18 (Table 1) probably favors the increased production of PAT in comparison with the herbal blends K23 and G13 without such additions, which had no detectable amounts of PAT (Figure 4). The average content of PAT in both herbal blends K14 and K18 with addition of elderberry fruit was 7.4 *µ*g/kg, while in both herbal blends G7 and K19—containing apple and chokeberry fruit—this value was nearly sevenfold increased up to 50.5 *µ*g/kg. In particular, we observed that adding sliced apple fruit, accompanied by addition of chokeberry and hibiscus flowers, led in the herbal blend K19 to a very high increase in PAT concentration up to 93.2 *µ*g/kg (Figure 1). Our finding could be quite reasonable since the PAT producing *Penicillium expansum* isolates, as confirmed by detecting the *patN* gene coding the key enzyme isoepoxydon dehydrogenase involved in the PAT metabolic pathway, has been recently identified in fresh chokeberry (*Aronia melanocarpa* (Michx.) Elliott) harvested in Bulgaria [33] and from the same pome fruit cultivated in Italy [34]. In addition, some *Penicillium commune* strains producing patulin-like compounds have been isolated from endophytic fungi of the semimangrove flowering tree of the beach hibiscus (*Hibiscus tiliaceus* L.) distributed in the tropical and subtropical coastal regions of China [35]. Recently, both *Penicillium* sp. and *Aspergillus* sp. mold strains were reported in the endopytic flora of the Krosor and Samyl industrial cultivars of elderberry (*Sambucus nigra* L.) freshly harvested in northern regions of Poland [36].

Depending on the type of apple variety and place of harvest, the content of PAT was in a range from 1.0 to 70.6 *µ*g/kg in Portugal and from to 8.8 to 417.6 *µ*g/kg in the USA [37]. In Portugal, samples of apples and quinces have been examined for the presence of PAT using a GC-MS method by Cunha et al. [30], who determined PAT in stored apples covered with 25% brown areas had an average content of 3.2 *µ*g/kg, but in apples covered with 75% brown areas, the PAT content was increased to 1500.0 *µ*g/kg. The same report showed that 100% of organic apple juice was contaminated with PAT at an average level of 8.9 *µ*g/kg, and 50% of conventional apple juice had 9.9 *µ*g/kg of PAT. In turn, the quinces with 25% and 75% brown areas were infected by PAT at an average level of, respectively, 4.9 and 118.3 *µ*g/kg [30]. For comparison, the average concentration of PAT in pineapples and seedless grapes in Pakistan were the highest, respectively, at 254.1 and 286.1 *µ*g/kg [14].

**Figure 5:**
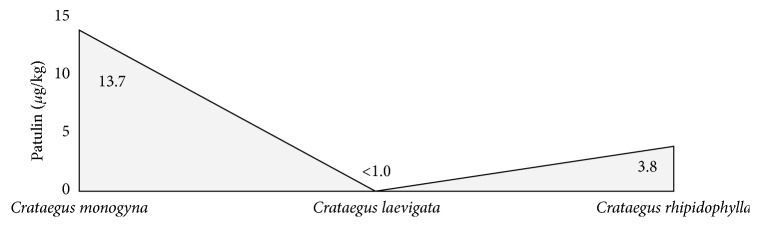
Distribution average content of PAT (*µ*g/kg) in the 41 studied commercial dietary supplements, herbal blends, and freshly harvested hawberry in relation to the identified genus species of *Crataegus* spp. (Tables 1 and 2).

In the samples of naturally dried hawberry G23, G24, and G25 harvested from *Crataegus laevigata* mature trees located in Ojcow (Poland, Malopolskie Province), the average PAT concentration was below the limit of detection (LOD = 1.0 *µ*g/kg), while in the samples G26, G27, and G28 with hawberry harvested from the *Crataegus rhipidophylla* mature trees in Ojcow, located in the southern part of Poland, the maximum PAT content was 9.5 *µ*g/kg. In this special case of samples, the average PAT content was increased to 3.8 *µ*g/kg in spite of the fact that both of these kinds of hawberries were processed and stored under the same drying conditions at +20°C (Figure 5).

Considering the accumulation of PAT in the hawberries obtained from the various hawthorn genus species, it can be seen that PAT was probably not biosynthesized in the hawberry obtained from the species of *Crataegus laevigata* (samples G23, G24, and G25; Tables 1 and 2 and Figure 5). Barad et al. [18] suggested that cultivars of apple trees (which like the hawthorn tree belong to the rose family, *Roseacea*) are an important factor influencing the inhibition of PAT biosynthesis. The Golden Supreme variety of apples can become completely rotten, showing visible mold growth in the exterior and interior of the fruit (incubated for 59 days at 20.5°C), but there were was no PAT accumulation detected during the analysis. On the contrary, the Fuji variety of apple analyzed after 80 days of incubation at 20.5°C resulted in a PAT concentration at a level of 5.9 *µ*g/kg. Additionally, in the McIntosh variety of apples, PAT was not detected after 80 days of incubation at 11.0°C [23]. These data and some results from our present study indicate that there is a need to extend future research to the content of PAT and the specific fungal species producing this mycotoxin in the hawberry of every individual genus species of the *Crataegus* genus.

Patulin (PAT) was not detected in the frozen hawberry harvested from the *Crataegus monogyna* mature hawthorn trees located in Fordon and Bartodzieje districts in Bydgoszcz (*n*=15, group III, Tables 1 and 2). It is well known that temperature has a principal influence on PAT biosynthesis and its production. A refrigeration temperature of +4.0°C does not prevent PAT accumulation in apple fruit, but dropping the temperature to +1.0°C reduces PAT production significantly [38]. However, an increase in the PAT concentration with a temperature drop from +20.0 to +4.0°C was not observed for all strains of *Penicillium expansum* [38]. Similar results were obtained by McCallum et al. [39] who observed that PAT concentration in apples was decreased with a temperature drop from +25.0 to +4.0°C. The results of Murillo-Arbizu et al. [40] indicated that PAT is stable over a period of 6 months at −20.0°C and there was no protein denaturation effect on the PAT content. These reference data suggest that, before the freezing process applied to hawberry is collected from the *Crataegus monogyna* trees, PAT production had not been initiated and that storage conditions of −20.0°C as applied in our study to the frozen hawberry successfully enabled the avoidance of biosynthesis of PAT in this kind of fruit.

**Figure 6:**
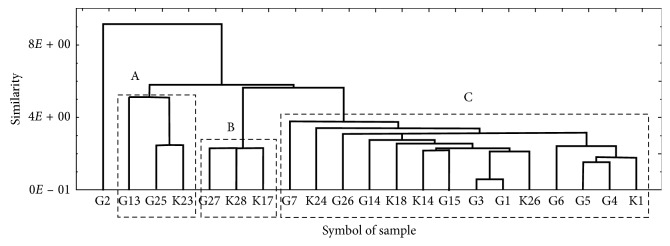
Dendrogram (classification tree) showing similarity of the studied commercial dietary supplements, herbal blends, and freshly harvested hawberries (groups I, IA, and II; Table 1) in view of determined PAT content, producer, province of origin, genus species of *Crataegus* spp., and composition (single-component versus multicomponent commercial products).

In Figure 6, the results of the hierarchical cluster analysis in the form of a dendrogram is presented in which the PAT-contaminated 19 products and samples with hawberry from groups I, IA, and II (Table 1) were classified into the three separate clusters: A, B, and C. In fact, an additional separate single element cluster is visible in Figure 6 in which the dietary supplement G2 with the highest average content of PAT (25.9 *µ*g/kg) was included. In clusters A and B, three studied hawberry products were included, while cluster C included fourteen products. Cluster A includes the analyzed hawberry products G13, G25, and K23 in which PAT was below the limit of detection, and all of these products originated from Malopolskie and Lubuskie provinces (Table 1). The hawberry-derived products K23 and G13 belong to the multicomponent herbal blends containing, respectively, 40 and 51% hawberry harvested from *Crataegus monogyna* and/or *Crataegus laevigata* trees but in unknown proportions in the final product. Sample G25 contained only the hawberry harvested from the wild tree of *Crataegus laevigata* (Table 1).

Cluster B in Figure 6 included three hawberry-derived products G27, K28, and K17 with nearly the same concentration of PAT, 15.7, 15.2, and 17.2 *µ*g/kg, respectively (Figure 4), and originated from Podlaskie and Lodzkie Provinces. These three commercial one-component dietary supplements were formulated exclusively with dried hawberry collected from the hawthorn tree species *Crataegus monogyna* and/or *Crataegus laevigata* (Table 1).

The third main cluster C in Figure 6 contained fourteen hawberry-derived products characterized by a lower average content of PAT in the range of 5.1 to 10.1 *µ*g/kg. In this cluster C, the dietary supplements G3, G1, and K26 characterized by their relatively low PAT contents in the range 5.7–8.3 *µ*g/kg originated in Wielkopolskie Province (western part of Poland) and were produced by the same manufacturer Kawon (Poland). In this cluster C, a second subgroup of analyzed products was very closely classified; i.e., dietary supplements G5, G4, and K1 were characterized by a slightly increased PAT content in the range 6.6–9.9 *µ*g/kg, and it originated from the same province (Lodzkie, central Poland) but a different manufacturer (Flos) (Poland).

**Figure 7:**
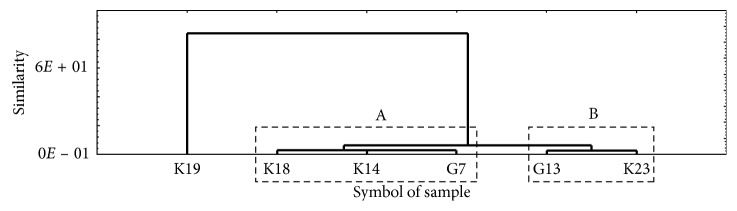
Dendrogram (classification tree) showing similarity of studied commercial multicomponent herbal blends (group IA, Table 1) in view of determined PAT content and individual ingredients.

In Figure 7, an additional separate dendrogram was calculated and constructed for the analyzed six multicomponent herbal blends (group IA, Table 1) considering the observed average PAT content, manufacturer, province of origin, genus, and species of *Crataegus* spp., and type of additional ingredients. A separate single element cluster is visible in this dendrogram in which herbal blend K19 with the highest content of PAT (93.2 *µ*g/kg) was included. In cluster A, were classified the three herbal blends K18, K14, and G7, which contained the highest whole fraction of various fruits such as the hawberry (up to 61%), elderberry (up to 18%), and apple fruit (Table 1). The average content of PAT in these three herbal blends was 7.5 *µ*g/kg, with a range of 5.8 to 9.0 *µ*g/kg (Figure 4). Flowers and leaves of various medicinal plants constituted a smaller proportion of the analyzed herbal blends (group IA, Table 1), but the herbal blends K18, K14, and G7 containing the addition of hibiscus and mallow flowers have been included in cluster A (Table 1).

Cluster B in Figure 7 included herbal blends G13 and K23 in which the average PAT content was below the limit of detection. The composition of these two herbal blends, in addition to hawberry, included hawthorn inflorescence (which is probably free of PAT), hibiscus flower, lemon balm leaf, lavender flower, horsetail herb, and small amounts of dog rose flower. We suggest that the high content of melatonin, selenium, and ascorbic acid and low-weight organic acids in these additional ingredients of both herbal blends G13 and K23 could protect them from the patulin-producing fungal strains [41, 42].

Until now, to our best knowledge, no study has reported on the content of PAT in commercial herbal blends containing hawberry harvested from the hawthorn trees species of *Crataegus monogyna* and/or *Crataegus laevigata*, which are used in the form of infusions and herbal teas in the prevention of hypertension and other cardiovascular diseases [25–28]. Consumption of apple fruit and apple juices and infusions of dietary supplements containing dried hawberry by adult humans could lead cumulatively to an increased risk of an excessive daily patulin intake (DPI) that can then be compared with the JECFA-WHO recommended provisional maximum tolerable daily intake (PMTDI) of PAT [22, 23]. Thus, the following criteria were adopted here for estimation of this DPI value by adult subjects suffering from cardiovascular diseases in Poland: mean body weight 70 kg, portion of 2.5 g of dried hawberry for preparation of infusion with 200 mL of boiling water (100°C) in time of 10 min, and consumption of three 200 mL glasses of mentioned earlier infusion per day as the quantity specified frequently by producers of hawberry-derived dietary supplements [41, 42] with an average content of PAT at 14.9 *µ*g/kg (as calculated from the 19 samples analyzed here that were contaminated by this mycotoxin). PAT is classified as the noncharged, polar, and strongly hydrophilic lactone-type compound (log *K*_ow_=−2.40, log *P*=−1.0) which indicates a very good solubility in water and common polar solvents [43], and thus near-complete 100% extraction of PAT from hawberry-derived products to their water infusions could be hypothesized. However, PAT is classified rather as the heat-resistant mycotoxin, especially in solutions with pH range 3.5/5.5, and its degradation levels of 18.8 and 26.0% after heat treatments for 20 min at 90 and 100°C, respectively, have been reported [44–47]. Similarly, pasteurization at 60/90°C for 10 s resulted in 18.8% reduction in PAT concentration observed in apple cider, but heating for 20 min at 70/80°C during the evaporation process resulted, respectively, in 9.4 and 14.06% degradation levels of PAT [45–47]. Thus, based on results of these previous reports [44–47], during home preparation of considered hawberry infusion, an approximate degradation level of PAT equal to 15% could be reasonably hypothesized.

With these criteria, the calculated average DPI was equal to 0.0014 *µ*g PAT (i.e., 1.4 ng PAT) per kg of body weight. This value represents 0.35% of the PMTDI recommended by JECFA-WHO as 0.4 *µ*g PAT per kg of body weight [23]. More than 70-fold higher values of daily patulin intake (DPI) have been reported for consumption of apple juices by infants, i.e., 26% of the PMTDI recommended by JECFA-WHO [40], and in cases of apple juice consumption by children, 24% of this PMTDI was reported by Baert et al. [22]. However, the overall chronic intake assessment of PAT in a Chinese population revealed that only 0.00004% of the abovementioned PMTDI (i.e., 16 pg per kg of body weight) was consumed via dried hawberry products [28]. This means that estimated average DPI for hawberry-derived products from Poland indicated the near ten thousand-fold increased the value compared to DPI calculated for the same Chinese hawberry products. Thus, despite the relatively low content of PAT in the hawberry-derived products from Poland and the protective action of dietary supplied green tea polyphenols, vitamin E, and apigenin against deleterious effects of PAT in humans [17], it is necessary to pay more attention to the fact that, in older humans, aging causes progressive degenerative changes in all cells and organs, thus reducing the tolerance of microbial toxins by the human body [48–50]. Especially, regular consumption of PAT-contaminated dietary supplements and foodstuffs by humans with reduced cell concentration of glutathione caused by inflammation, hypoxia, or enzyme polymorphism could lead to the increased risk of PAT-related genotoxicity and severe damage of key organs like kidney, intestinal tissue, and immune system [51].

## 4. Conclusions

A sensitive, reproducible, and rapid UHPLC-MS/MS method was described in this paper for determination of patulin (PAT) concentration in commercial dietary supplements and herbal blends containing dried hawberry (*n*=20). The results show that an alarming proportion of 90% (*n*=18) of the analyzed commercial products from Poland was contaminated with PAT. One of the studied herbal blends (K19) exceeded by almost twice the acceptable maximal limits for PAT in fruit products recommended by the WHO, i.e., 93.2 vs 50.0 *µ*g/kg. It could be hypothesized that alone or combined with apple, chokeberry, elderberry, or hibiscus flower while not sufficiently controlling the microbial quality in these hawberry-based herbal blends could lead to an increased content of PAT in these types of commercial marketed medicinal products. Moreover, PAT was found at an average concentration of 11.4 ± 5.5 *µ*g/kg and range of 5.7 to 25.9 *µ*g/kg in every analyzed dietary supplement (*n*=14) containing only dried hawberry collected from the hawthorn species *C. monogyna* and/or *C. laevigata*. In our study, PAT was not found in the naturally dried hawberry at +20.0°C that was harvested from *Crataegus laevigata* (samples G23, G24, and G25 in Table 1), while an average content of PAT of 3.8 *µ*g/kg was found in the naturally dried hawberry obtained from *Crataegus rhipidophylla* (samples G26, G27, and G28) harvested in the national park area of Ojcow (Poland). Similarly, PAT was not detected in the frozen hawberry at −20.0°C (*n*=15) collected from the mature wild hawthorn trees *Crataegus monogyna* in the recreational forest area of Bydgoszcz (Poland). In conclusion, we suggest that there is an urgent need to intensify and extend the analytical control related to the presence of PAT in freshly harvested, stored, and processed hawberry and other fruit components used as ingredients in dietary supplements and herbal blends that are commonly consumed by a broad range of adult subjects with serious cardiovascular disorders.

## Figures and Tables

**Table 1:** Characteristic data of the studied 41 products and samples containing hawberry (hawthorn fruit).

No.	Sample	Species of *Crataegus* spp.	Producer	Province of Poland	Composition
*Group I: single-component commercial dietary supplements*					
1	K1	*C. monogyna*/*C. laevigata*	Flos	Lodzkie	Hawberry (hawthorn fruit)
2	K17		Dary natury	Podlaskie	
3	K24		Natur-vit	Swietokrzyskie	
4	K26		Kawon	Wielkopolskie	
5	K28		Dary natury	Podlaskie	
6	G1		Kawon	Wielkopolskie	
7	G2		Kawon	Wielkopolskie	
8	G3		Kawon	Wielkopolskie	
9	G4		Flos	Lodzkie	
10	G5		Flos	Lodzkie	
11	G6		Dary natury	Podlaskie	
12	G27		Flos	Lodzkie	
13	G14		Skarby natury	Lubuskie	
14	G15		Internet sale	No data	

*Group IA: multicomponent commercial herbal blends*					
15	K23	*C. monogyna*/*C. laevigata*	Herbapol	Malopolskie	Hawberry (40%), lemon balm leaf (30%), lavender flower (15%), horsetail herb^x^
16	G7		Bifix	Lodzkie	Hawberry (40%), apple fruit^x^, chokeberry fruit^x^, hibiscus flower^x^, citric acid^x^
17	G13		Malwa	Lubuskie	Hawberry (51%), hibiscus flower^x^, hawthorn inflorescence^x^, rose hip^x^
18	K14		Herbapol	Malopolskie	Hawberry (60%), elderberry fruit (18%), hibiscus flower (17%), mallow flower (5%)
19	K18		Herbapol	Malopolskie	Hawberry (61%), elderberry fruit (18%), hibiscus flower (17%), mallow flower (4%)
20	K19		Bifix	Lodzkie	Hawberry (40%), apple^x^, chokeberry fruit^x^, hibiscus flower^x^, concentrate of chokeberry fruit^x^, citric acid^x^

*Group II: naturally dried at 20°C, freshly harvested hawberry*					
21	G23	*C. laevigata*	Ojców^a^	Malopolskie	Hawberry
22	G24	*C. laevigata*	Ojców^a^	Malopolskie	Hawberry
23	G25	*C. laevigata*	Ojców^a^	Malopolskie	Hawberry
24	G26	*C. rhipidophylla*	Ojców^b^	Malopolskie	Hawberry
25	G27	*C. rhipidophylla*	Ojców^a^	Malopolskie	Hawberry
26	G28	*C. rhipidophylla*	Ojców^a^	Malopolskie	Hawberry

*Group III: frozen at −20°C, freshly harvested hawberry*					
27	F1	*C. monogyna*	Fordon 1^c^	Kujawsko-Pomorskie	Hawberry
28	F2		Fordon 1^c^		
29	F3		Fordon 1^c^		
30	F4		Fordon 1^c^		
31	F5		Fordon 1^c^		
32	F6		Fordon 1^c^		
33	F7		Fordon 1^c^		
34	F8		Fordon 1^c^		
35	F9		Fordon 1^c^		
36	F10		Fordon 1^c^		
37	F11		Fordon 1^c^		
38	F12		Fordon 2^d^		
39	F13		Fordon 2^e^		
40	F14		Fordon 2^f^		
41	F15		Bartodzieje^g^		

*Notes*. ^x^Not defined content. ^a–g^Geographical coordinates for location: ^a,b^50°12′37.4″N, 19°48′49.0″E (radius of 2 km); ^c^53°09′52.7″N, 18°09′07.3″E (radius of 200 m); ^d^53°09′46.1″N, 18°09′50.4″E; ^e^53°09′36.9″N, 18°09′59.9″E; ^f^53°09′22.9″N, 18°09′13.2″E; ^g^53°07′29.5″N, 18°02′29.4″E.

**Table 2:** Content of patulin (PAT) in the 41 analyzed products and samples with hawberry from Poland.

Products type	Group^x^	Percentage of PAT positive samples	Average PAT content (*µ*g/kg)	Median PAT content (*µ*g/kg)	Range of PAT content (*µ*g/kg)
Commercial single-component dietary supplements (*n*=14)	I	14/14 (100%)	11.4 ± 5.5^a^	9.5	5.7/25.9
Commercial multicomponent herbal blends (*n*=6)	IA	4/6 (67%)	15.7 ± 31.6^b^	6.8	<LOD/93.2
Naturally dried at 20°C freshly harvested (*n*=6)	II	1/6 (17%)	2.4 ± 3.4^d^	1.1	<LOD/9.5
Frozen at −20°C freshly harvested (*n*=15)	III	0/15 (0%)	<LOD^c^	<LOD	<LOD

*Note*. LOD = 1.0 *µ*g/kg; ^x^group of analyzed samples as specified in Table 1; ^a–d^ statistically significant differences in content of PAT (*p* < 0.05).

## Data Availability

The data used to support the results of this study are included within the article.
